# Supplementary material for: Patterns of Intron Gain and Loss in Fungi
Source: PLoS Biol. 2004 Nov 30;2(12):e422. doi: 10.1371/journal.pbio.0020422 (PMC532390; doi:10.1371/journal.pbio.0020422)
Supplement: Table S1 — Also available at http://genes.mit.edu/NielsenEtAl/. (4.3 MB ZIP). [file pbio.0020422.st001.zip › NielsenEtAl/html/1344.html]

AN6487.1.NCU00338.1.MG06327.1.FG05471.1


```
 CLUSTAL W (1.82) Multiple Sequence Alignments - Introns Inserted


Sequence 1: AN6487.1	454 aa
Sequence 2: FG05471.1	467 aa
Sequence 3: MG06327.1	474 aa
Sequence 4: NCU00338.1	394 aa
Alignment Length: 516 aa
Number Identitical Residues: 111 aa
Alignment Score (without introns) 6645


MG06327.1 	MRLIASLALAASLASSI~TNGSTIPRRASGTPAAPRVIGLETERQHIPNPLERDRLRR--
NCU00338.1	-----------------~------------------------------------------
FG05471.1 	MKPTYSTLFLSLLASTE~A----ISLHKREHGLEPRVMSVPIQRRQIDNPLAHDRKRLNR
AN6487.1  	------MLRVGFDASTN2---------------------------EIPDPVARDRRRR-K
          	          .  :::                                ..   .      

MG06327.1 	-RAAVMATLDNE~QTLYFVNVSIGTPPQKLRLHLDTGSSDLWVNTPD~SKLCSVSSQPCR
NCU00338.1	------------~--------------------------------MS2SILCQDEDKPCE
FG05471.1 	RAGTVNVGIDNE0QSLYFLNASIGTPPQNFRLHLDTGSSDLWVNSVN~SELCDTHANICA
AN6487.1  	RDQTVAQLIDNE0ETLYFCNLTVGTPGQSLRLILDTGSSDLWCNAAN~STLCSSPKDPCR
          	   :     ... .:    . : .:. ..     .:.::.   .: . * **.   . * 

MG06327.1 	FAGTFSANSSSTYQYINSVFNISYVDGSGANGDYVSDMVTVGNTKIDRLQFGIGYTSSSA
NCU00338.1	YSGTYLANDSSTYEYISNHFDIKYVDGSGARGDYASDTFTIGNTKLNRLQFGIGYSSTNA
FG05471.1 	ESGLYNANKSSTYEYVNSGFNISYADGSGASGDYVTDTFRMGEVSIKDLQFGIGYITSDN
AN6487.1  	ISGSFDPSSSSSYSYISSDFNITYADGTGAAGDYATDTVSIGGATIKDFQFGIGYTSSSA
          	 :* : ...**:*.*:.. *:*.*.**:** ***.:* . :* ..:. :****** ::. 

MG06327.1 	Q~GILGVGYEANEVQVGRAQLKPYRNLPSRMVEEGLIASNAYSLYLNDLQSNKGSILFGG
NCU00338.1	Q~GLLGIGYTLSEVQT-RAGLPAYNNLPAQMVADGLINSNAYSIWLNDLDALTGTILFGG
FG05471.1 	E~GVIGIGYTSNEAVVDQPDPEFYKNMPARLASDGVIASNAYSLYLDDLESATGKILFGG
AN6487.1  	E1GVLGIGYPSNEVQVARYGDDAYPNLPRFLMQNGFVQSSAYSLWLNDLEANTGSILFGG
          	: *::*:**  .*. . :     * *:*  :  :*.: *.***::*:**:: .*.*****

MG06327.1 	IDTEQYTGTLQTVPIQP-NGGRMAEFLITLTSVSLTSAS--------------IGGDKLA
NCU00338.1	VDAAKYEGDLLTLPVQTPEKGTYKNLMVTMTGLSLSQSQSSSSDKGNGDDTTQISKDNLA
FG05471.1 	VDEQHFIGDLVTVPIMK-INDEYSEFYVKLQSINSGSE---------------IVGEGLD
AN6487.1  	VDTEKYRGDLQTLPIQT-VNGEYSELIIALTGVSLDTEARKHT----------VSSNALP
          	:*  :: * * *:*:     .   :: : : .:.      . :          :  : * 

MG06327.1 	LAVLLDSGSSLTYLPDDIVKNMYSAVGAQYDS---NEGAAYVPCSLARDQANSLTFSFSG
NCU00338.1	LAVLLDTGSTLSYLPSELVKPLYDAIGIEYITDPDGKVDGYAPCHLMSSSQS-VMFSFSS
FG05471.1 	LGVVLDSGSTLTYLPSSVTDSIYQLVGADYEE---GQTTAYVPCDLANQGGN-LTFKFTS
AN6487.1  	AAVLLDSGSSLSYLPDSIAEKIYDDLRISYEP---STGAGYAPCSLARQNIN-VTFTFSS
          	 .*:**:**:*:***..:.. :*. :  .*     .   .*.** *  .  . : *.*:.

MG06327.1 	I-PIVVPMNELVLDLVTSNGRRPSFRNGVP-ACLFGVAP-AGKGTNVLGDTFLRSAYVVY
NCU00338.1	PLQIAVPMNELIVNR-TFHGKLPRMPDGVTDACIFGIQERNGTGANTLGDTFLRSAYVVF
FG05471.1 	PAEITVPLSELILDFTDITGRQMSFTNGQA-ACSFGIAP-STSQVSILGDTFLRSAYVVF
AN6487.1  	P-EIAVGIDELIIDAGDLR-----FSNGER-ACIFGLVP-AGDNTAVLGDTFLRSAYVVY
          	   *.* :.**:::          : :*   ** **:       .  ************:

MG06327.1 	DLENNAISLAQTSFNATKSNVKEIGKGSNPVPGAVAVSQPVAATSGLSQNGGNR------
NCU00338.1	DLDNNEISMAQTRFNATATDLKEIKKGKGGVPGAKAVENPVEATSGLSGNEGG-------
FG05471.1 	DLDNNEISLAQSNFEATGSHILEISKGKNAVPSATGSDGPQ--SSGSENAAGS-------
AN6487.1  	DLTNNEISIAKTNFNSTKSNILEIGTGSDAVPGATKVSHPV--TSVVADGSGSRIGAPTN
          	** ** **:*:: *::* :.: ** .*.. **.*   . *   :*      *.  .:.:.

MG06327.1 	---SGSGAIARAVPTL-------LLVGGIFSGSLLTLF--
NCU00338.1	---IYVNGAACELNVG------MGMAWGLLVGATMVVLGL
FG05471.1 	---LSPLGSTGAVSIL---------AGAMALTFAWFLI--
AN6487.1  	TEDIVPSASTGAAVVLGRSTISPVLVGAAALGYMFAF---
          	:..    . :      . :: :   . .        .
```
